# Novel kinase platform for the validation of the anti-tubercular activities of *Pelargonium sidoides* (Geraniaceae)

**DOI:** 10.1186/s12896-020-00643-w

**Published:** 2020-09-29

**Authors:** V. Lukman, S. W. Odeyemi, R. L. Roth, L. Mbabala, N. Tshililo, N. M. Vlok, M. J. B. Dewar, C. P. Kenyon

**Affiliations:** 1 Department of Life and Consumer Sciences, College of Agriculture and Environmental Sciences, University of South Africa, Johannesburg, 1709 South Africa; 2 Council for Scientific and Industrial Research, Pretoria, South Africa; 3 DST-NRF Centre of Excellence for Biomedical Tuberculosis Research, South African Medical Research Council Centre for Tuberculosis Research, Division of Molecular Biology and Human Genetics, Faculty of Medicine and Health Sciences, Stellenbosch University, Cape Town, 7505 South Africa; 4 Proteomics Spectrometry Unit, Central Analytical Facility, University of Stellenbosch, Private Bag X1, Matieland, Stellenbsoch, 7600 South Africa

**Keywords:** *Mycobacterium tuberculosis*, Kinases, Anti-tubercular, Target identification

## Abstract

**Background:**

*Pelargonium sidoides* is an important traditional medicine in South Africa with a well-defined history of both traditional and documented use of an aqueous-ethanolic formulation of the roots of *P. sidoides* (EPs 7630), which is successfully employed for the treatment of respiratory tract infections. There is also historical evidence of use in the treatment of tuberculosis. The aim of this study was to develop a platform of *Mycobacterium tuberculosis* (Mtb) kinase enzymes that may be used for the identification of therapeutically relevant ethnobotanical extracts that will allow drug target identification, as well as the subsequent isolation of the active compounds.

**Results:**

Mtb kinases, Nucleoside diphosphokinase, Homoserine kinase, Acetate kinase, Glycerol kinase, Thiamine monophosphate kinase, Ribokinase, Aspartokinase and Shikimate kinase were cloned, produced in *Escherichia coli* and characterized. HPLC-based assays were used to determine the enzyme activities and subsequently the inhibitory potentials of varying concentrations of a *P. sidoides* extract against the produced enzymes. The enzyme activity assays indicated that these enzymes were active at low ATP concentrations. The 50% inhibitory concentration (IC_50_) of an aqueous root extract of *P. sidoides* against the kinases indicated SK has an IC_50_ of 1.2 μg/ml and GK 1.4 μg/ml. These enzyme targets were further assessed for compound identification from the *P. sidoides* literature.

**Conclusion:**

This study suggests *P. sidoides* is potentially a source of anti-tubercular compounds and the Mtb kinase platform has significant potential as a tool for the subsequent screening of *P. sidoides* extracts and plant extracts in general, for compound identification and elaboration by selected extract target inhibitor profiling.

## Background

The prevalence in tuberculosis (TB), together with the recent increase in the incidence of multidrug-resistance (MDR) cases, has led to the search for new drug targets and new drugs that are effective against Mtb. TB is often a fatal disease, and one that poses a global threat to human health [5, 6]. Globally, infection associated with TB is second only to HIV/AIDS as the greatest killer due to a single initiating infectious agent [25]. Out of the 9 million people infected with TB in a year, 3 million are left untreated, acting as a reservoir for further infection. Many of these 3 million untreated cases live in poverty with minimal access to healthcare. Over 95% of TB cases and deaths are in developing countries such as South Africa, often where the percentage of HIV/AIDS co-infection is high, and therefore occurs in individuals with compromised immune systems (WHO, 2020) [25].

TB is caused by various strains of *Mycobacterium tuberculosis*, commonly affects the lungs, and is transferrable from person to person through the air. Mtb is an intra-cellular parasite normally residing in the human macrophages, where its survival and growth depends on complex networks of the attenuation of macrophage activity by the Mtb bacilli. Mtb has not only developed a number of mechanisms to evade onslaught from such host macrophage immune responses as reactive oxygen and nitrogen species, but it has also evolved a metabolism that has allowed it to survive in this very specialized niche environment [27]. It has become evident that one of the mechanisms to arrest the progression of Mtb into full-blown infection is a multi-targeted approach [23]. The key question which arises is, “Can a single plant extract be used to screen multiple targets as a means of identifying novel synergistic anti-infective properties?” Medicinal plants have been used for the treatment of several diseases and the plant extract identified for analysis was that from *Pelargonium sidoides,* as this plant has a 200 year documented history of ethnobotanical use in the treatment of tuberculosis and other infections [1, 2, 12, 13, 15, 18, 20]. This includes well documented reports in literatures that suggest the medicinal properties and efficacy of *P. sidoides* against *M. tuberculosis* and other bacterial infections [16, 9, 17, 22]*.* The mechanisms and targets of this anti-tubercular activity are, however, not defined. The aqueous-ethanolic extract of *P. sidoides* (EP® 7630) roots have been used to treat bacterial infections, as well as to induce the production of the pro-inflammatory cytokines TNF-α and IL-6 in human blood human immune cells, alleviating symptoms associated with acute bronchitis [12, 13]. This extract is licensed in Germany as herbal medicine for the treatment of upper respiratory tract infections [26].

This investigation was therefore set up to target a functionally diverse range of Mtb kinase enzymes as a mechanism of identifying potential Mtb drug targets using the complexity found in medicinal plant extracts as the source of chemical diversity. It was also decided to select a range of Mtb kinases which, where possible, do not occur within mammalian biochemistry. Kinases have been classified into 25 families of homologous proteins, with the families assembled into 12 fold-groups based on the similarity of their structural folds [3, 4]. It has further been demonstrated that each of the 12 fold-groups has a distinct phosphoryl transfer mechanism [11], and it was therefore decided to select the kinase enzyme targets from 6 of the 12 fold-groups, thereby representing 6 distinct phosphoryl transfer mechanisms. The six identified phosphoryl transfer mechanisms all have distinct ATP binding motifs. The selection of these kinases should therefore identify different classes’ compounds capable of binding ATP. The enzymes selected are crucial for the metabolism and survival of Mtb. As relatively large number of enzymes was to be comparatively simultaneously assessed it was envisaged to keep the enzyme purification and assays as simple as possible.

The eight specific Mtb kinases targeted are Nucleoside diphosphokinase (NDK, EC 2.7.4.6), Homoserine kinase (HSK, EC 2.7.1.39), Acetate kinase (AK, EC 2.7.2.1), Glycerol kinase (GK, EC 2.7.1.30), Thiamine monophosphate kinase (ThiL, EC 2.7.4.1), Ribokinase (RBKS, EC 2.7.1.15), Aspartokinase (AsK, EC 2.7.2.4), and Shikimate kinase (SK, EC 2.7.1.71) [7, 10, 14, 21]. The kinases were expressed in *Escherichia coli,* purified and the activity determined through HPLC-based assays. The inhibitory properties of a *P. sidoides* extract were then investigated against the characterized kinases.

## Results

The enzymes were expressed in *E. coli* with His-tags to facilitate the purification of these enzymes, and their functionality was validated by determining their enzyme activity before carrying out the *P. sidoides* inhibitory experiments.

### Cloning, expression and purification of enzymes

The Mtb kinase genes were PCR-amplified from *M. tuberculosis* H37Rv genomic DNA, yielding amplicons of 415, 952, 1162, 1558, 1006, 919, 1268 and 520 bp for *ndkA* (nucleoside diphosphate kinase)*, ThrB* (homoserine kinase)*, ackA* (acetate kinase)*, glpK* (glycerol kinase)*, thil* (thiamine monophosphate kinase)*, rbks* (ribokinase)*, ask* alpha and *ask* beta (aspartokinase) and *aroK* gene (shikimate kinase), respectively. These were subcloned into the selected plasmids and confirmed via sequencing.

**Fig. 1:**
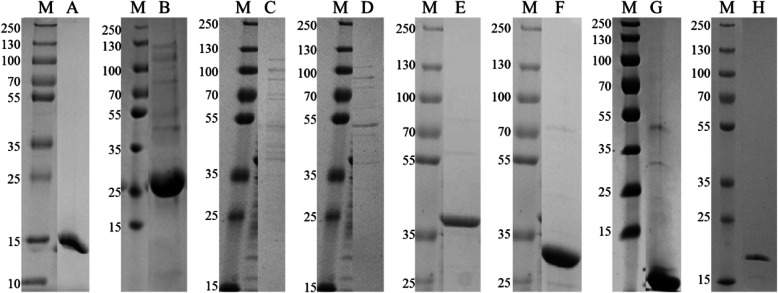
SDS-PAGE gels of the Mtb his-tagged kinases purified from *E. coli* BL21 (DE3). M represents the molecular mass marker (PageRuler™ Plus Pre-stained Protein Ladder, Thermo Scientific, USA) with the sizes of the bands indicated to the left of the gels in kDa. **a** NDK, 14,4 kDa. **b** HSK, 33.4 kDa. **c** AK, 43.7 kDa. **d** GK, 58.2 kDa. **e** ThiL, 36.4 kDa. **f** RBKS, 32.3 kDa. **g** AsK alpha, 44.6 kDa and AsK beta, 18 kDa. **h** SK, 20.7 kDa

All enzymes were expressed in *E. coli* BL21 (DE3) subsequent to IPTG induction. Following nickel-affinity purification using either native or denaturing means, the proteins was verified by SDS-PAGE as outlined in Fig. 1 and in Additional file 1 for the detailed break-down of the fractions obtained. Acetate kinase (AK) and glycerol kinase (GK) were both well expressed however the final eluate yielded lower levels of protein. They were both however still used in the screening as sufficiently high levels of enzyme activity were obtained. It was decided to keep these enzymes in the screen panel as one of the primary aims of setting up the screen panel was to assess if the panel may be used to identify compound target selectivity from mixtures such as plant extracts. It is envisaged that a higher fidelity secondary screen will be set up, using purer enzyme, once the target has been identified. The secondary screen will be used for compound identification.

### Enzyme activity assays

**Fig. 2:**
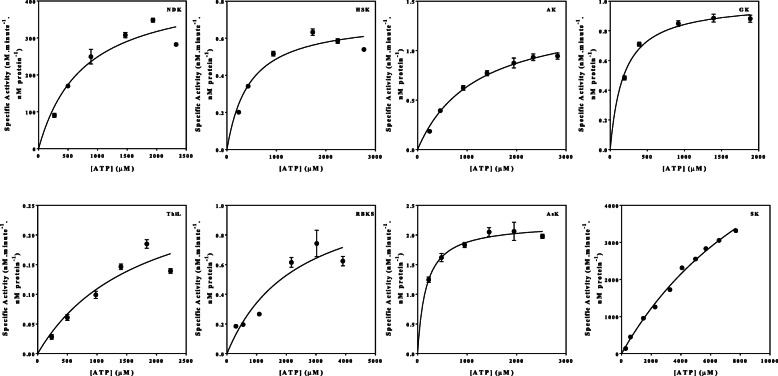
The effect of the ATP concentration on the specific enzyme activities of the purified kinase enzymes

The effect of the ATP concentration on the steady state specific activity of the *M. tuberculosis* kinases was expressed over a concentration gradient of ATP (Fig. 2). As the enzymes were recombinantly expressed in *E. coli*, the specific activity of the individual enzymes referred to throughout the document is the recombinant specific activity as the enzymes were not obtained from their native host. The best-fit to the data was obtained for the specified kinetic model using the non-linear regression algorithms as outlined using the GraphPad Prism® 5 software. The variation in the maximum specific enzyme activity for each enzyme was vast, ranging from approximately 0.14 nM/minute/nM protein for AsK to in excess of 3000 nM/minute/nM protein for SK, indicative of the great variation in binding affinity for ATP of the selected enzymes.

### *P. sidoides* inhibitory assay

**Fig. 3:**
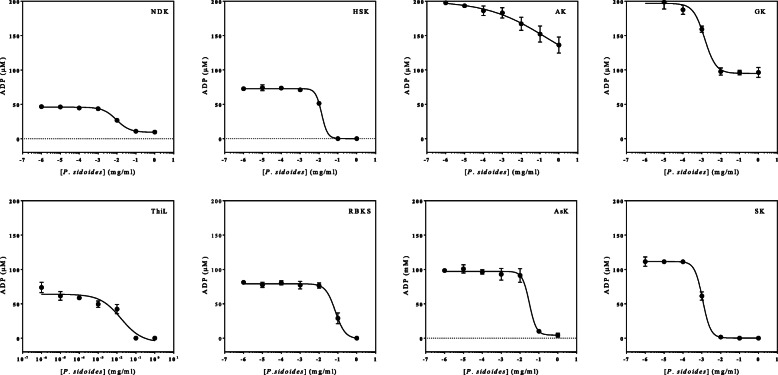
The inhibitory activities of P. sidoides against the purified kinases

The inhibitory activities of various dilutions of *P. sidoides* extracts were concentration-dependent in all the kinases except HSK, RBKS and AsK (Fig. 3). No inhibition was observed at 1 × 10^− 6^ mg/ml of *P. sidoides* on ThiL and RBKS kinases. The most susceptible enzymes to *P. sidoides* extract were SK and GK with the lowest IC_50_ values of 1.17 mg/ml and 1.4 mg/ml, respectively, when compared to other kinases (Table 1).

**Table 1:** Kinases and their respective IC50 values derived from the dose-response curves

Kinase	IC_50_ value (mg/ml)
NDK	9.28
HSK	13.72
AK	212.3
GK	1.40
ThiL	16.01
RBKS	73.42
AsK	31.16
SK	1.17

The validation for presence of the enzymes to complement the enzyme functionality data was demonstrated using peptide mass spectroscopy (Table 2). The data and the diagnostic mass fragmentation patterns for selected peptides are outlined in Additional file 1.

**Table 2:** Kinases and their respective MS validation

Kinase	Peptide	Probability (%)	Unique Peptides	Unique Spectra
NDK	(R)KGLTIAALQLR(T)	100	11	18
HSK	(K)GFAVTELTVGEAVR(W)	100	65	100
AK	(K)MLAEDGIDLQTcGLVAVGHR	99.7	34	47
GK	(R)DQLGIISGAAQSEALAR(Q)	99.7	55	68
ThiL	(R)TVVSTDMLVQDSHFR(L)	100	3	3
RBKS	ND			
AsK	(R)cVEYARRHnIP(V)	99.7	3	3
SK	ND			

*ND* not detected

## Discussion

**Fig. 4:**
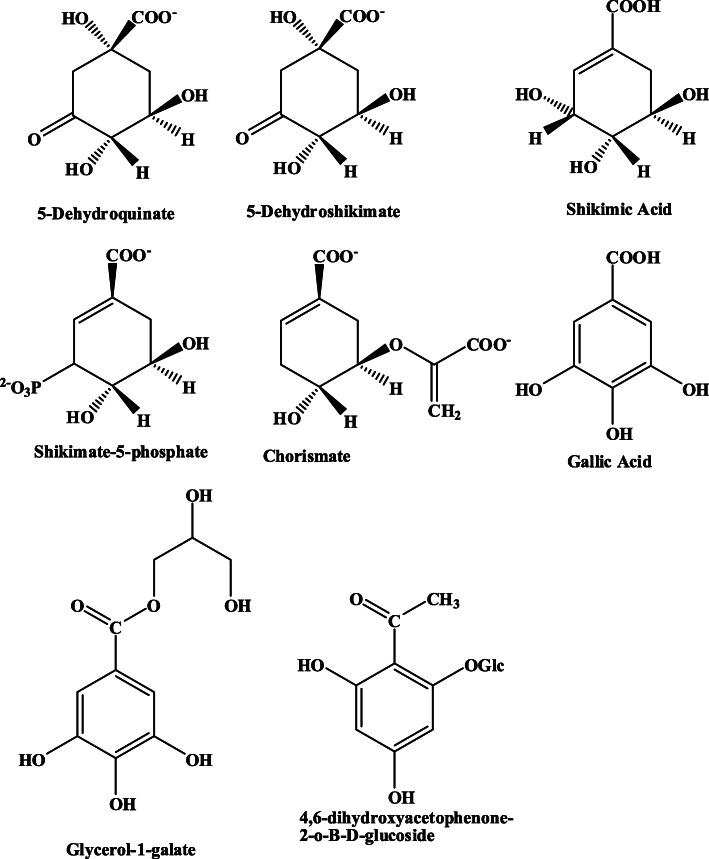
Structural similarity between gallic acid and a few o-galloyl derivatives which are components of P. sidoides and shikimic acid, the substrate to SK

A range of kinases was selected for screening, based on representing some of the 12 fold-groups that have distinct phosphoryl transfer mechanisms, with a total of 6 distinct phosphoryl transfer mechanisms represented in the 8 enzymes used in this study [11]. The purification of the proteins was challenging, but a variety of different techniques were investigated in order to acquire sufficient amounts of relatively pure protein (as estimated by PAGE) to run all assays simultaneously as part of a preliminary medicinal plant extract screen. The presence of each kinase was demonstrated by enzyme activity, SDS-PAGE and/or MS analysis. The competitive inhibition of the kinase reaction may manifest either as inhibiting the binding of ATP or by the inhibition of the binding of the enzyme substrate that is to be phosphorylated. As these enzymes were selected based on the fact that they all have distinct phosphoryl transfer reactions as well as distinct substrates, it was envisaged that the plant extract may demonstrate a significant variation on the IC_50_s obtained. This was found to be the case, with SK and GK having IC_50_ values of 1.17 mg/ml and 1.4 mg/ml, respectively, with all the other enzymes having IC_50_s of at least one order of magnitude higher. Clearly, the kinases selected could be used in a primary selection to identify targets for a plant extract. The selected enzymes could then be used in a more stringent secondary screen to identify the active compounds. What is significant is one of the major active ingredients of *P. sidoides* is gallic acid, which is a shikimic acid mimic (the substrate of SK) (Fig. 4). Gallic acid and a range of *O*-galloylated compounds have been demonstrated to be present in the extracts of *P. sidoides* [12]. SK has been identified as being a potential target to develop antimicrobial agents for Mtb [8, 19]. An associated species of *Pelargonium* used in South Africa for medicinal purposes is *Pelargonium reniforme*. *P. reniforme* produces *O*-galloylated glycerol (glycerol-1-gallate) which could be a potential bi-functional inhibitor of both SK and GK. If only low levels of glycerol-1-gallate are synthesized in *P*. *sidoides*, however if the binding constants of glycerol-1-gallate for SK and GK is high enough inhibition will still occur and probably synergistically. As the enzymes selected are all kinase enzymes it is realistic to believe that one of the binding mechanisms is in the kinase ATP binding site. These results clearly demonstrate that this platform of Mtb kinase enzymes can be used as a primary selection strategy for the identification of active ingredients in plant extracts that allows for the stratification of the inhibition of Mtb kinase enzymes and the validation of the extracts potential medicinal properties.

The traditional use of the plant root of *P. sidoides* was for a wide range of ailments including tuberculosis (for well researched review on the ethnobotanical and medicinal use of *P. sidoides* and other Pelargonium specices see reference [2];). *P. sidoides* forms the basis of Charles Henry Stevens secret cure for tuberculosis, “Stevens Cure” and was called “Umckaloabo”. Modern aqueous-ethanolic formulation of the roots of P. sidoides (EP® 7630) has been successfully employed for the treatment of ear, nose and throat disorders as well as respiratory tract infections [12, 13].

The data exhibited only moderate direct antibacterial capabilities against a spectrum of Gram-positive and Gram-negative bacteria, although convincing data was provided in support for the improvement of immune functions at various levels, hence, validating the medicinal uses of EP® 7630. The concentrations and nature of the active compounds in the extract are unknown. This data however, provided support to validate the medicinal use of *P. sidoides*. However, the remedial effects are not yet associated with mechanistic structure and function analyses and therefore further investigations are required in order to study the functional relationships between the *O*-gallolylated compounds and SK and GK. Phytochemical studies show the presence of a large number of other secondary metabolites in the plant, such as tannins, coumarins, phenolic acids, phenylpropanoid derivatives and other chemical constituents [12, 17]. This identified platform of Mtb kinases could serve as a screen to allow for mechanistic studies to be carried out on ethnobotanical plant extracts. The traditional use of *P. sidoides* and the present enzyme results indicate the potential use of this kinase platform to directly relate traditional use to target mechanistic investigations thereby identifying the potential drug target. These data should eventually contribute to evidence-based traditional medicines.

## Conclusions

In conclusion, selected Mtb kinases were successfully expressed in *E. coli* and purified and validated by SDS-PAGE, enzyme activity and/or MS spectroscopy. The enzyme functionality was validated through the enzyme activity of the purified proteins, and the effect of a *P. sidoides* plant extract on their activities was determined. The most susceptible enzymes tested were SK and GK, with the lowest IC_50_ values. This suggests that both SK and GK could be used as targets through which *P. sidoides* extracts could be characterized in terms of the specific chemistry of the inhibitors. As these two enzymes have different phosphoryl transfer mechanisms is it probable that different classes of compounds will be selected. The biosynthesis of the aromatic amino acids occurs via chorismate, the precursor to which is shikimate. As mammals do not have the biochemistry for the synthesis of chorismate or any of its intermediates, SK is a good validated target for Mtb. The human essential amino acids, tyrosine, tryptophan and phenylalanine are all synthesized using chorismate as the precursor. *P. sidoides* contains a broad range of O-galloylated compounds all of which are potential inhibitors of SK and in the case of glycerol-1-gallate, GK. Having identified the potential targets for *P. sidoides* inhibition SK and GK will therefore serve as a good screen for compound identification and validation from *P. sidoides* extracts.

## Methods

### Materials

H37Rv genomic DNA was received from Professor Ian Wiid, University of Stellenbosch, South Africa. The Mtb *aroK* gene (encoding Shikimate Kinase) in pET15-b was received from the laboratory of Chris Abell, University of Cambridge. All other genes were cloned in-house as outlined in section 5.3. All PCR reagents were from Kapa Biosystems (KAPA HiFi for gene amplification and KAPA 2G Fast for screening), and cloning materials were purchased from Epicentre Technologies, USA (Fast-Link™ DNA Ligation Kit) or Zymo Research, USA (Zyppy™ Plasmid Miniprep kit and Zymoclean™ Gel DNA Recovery Kit). Oligonucleotides for gene amplification were obtained from IDT Inc. (USA). All other chemicals used were at least analytical grade and were obtained from Sigma-Aldrich.

### Plant material

The *P. sidoides* fresh plant material was supplied by the Natural Plants and Agroprocessing (NPA) division of CSIR Biosciences through the CSIR Enterprise Creation for Development (ECD) division, Pretoria, South Africa. In an endeavor to limit the environmental destruction due to the uncontrolled wild harvesting of plants the ECD of the CSIR has facilitated the cultivation of a number of important ethnobotanical plants of known high usage, *P. sidoides* being one of them. The plants used were cultivated by Rodene Nursery, (ECD Sample number, ECD-MP-0252; Extract number, PEL-223-48448A). All plant taxonomy at the ECD was done in conjunction with South African National Biodiversity Institute (SANBI) which forms part of the The Plant List protocol.

The primary pre-processing post agricultural harvesting involved washing of the biomass (stalks and leaves cut into smaller pieces) prior to drying at 40-50 °C, over 3–5 days. The material was then milled using a hammer mill before carrying out the batch extraction process. The extraction was carried out in a glass jacketed percolation column. The biomass material was held in place within the column using mutton cloth. The percolation process was then carried out over 7 h. The extraction solvent used was ethanol (43% v/v EtOH, 1.25 L) and effective dried raw material loading (0.25 kg). The solvent was re-circulated through the column reactor over the period of 7 h. The solvent is pumped in at the top of the percolation column and allowed to diffuse through the column under gravitational force. The flow rate was estimated to be 36 ml/min. The percolator column dimensions (out diameter 11 cm, column length ~ 32 cm). The recovered filtrate (ex. percolation) was then collected and ethanol removed using a Buchi evaporator (40-60 °C, − 85 kPa over 1–2 h). The extract yield was 16.917 g (yield 6.76% m/m). The crude extract was stored at 4 °C.

### Cloning of the kinase genes from *M. tuberculosis*

The genes that were cloned were *ndka* (nucleoside diphosphate kinase NDK; Rv2445c), *thrB* (homoserine kinase HSK; Rv1296), *ackA* (acetate kinase AK; Rv0409), glpK (glycerol kinase GK; Rv3696c), *thiL* (thiamine monophosphate kinase Thil; Rv2977c), *rbks* (ribokinase RBKS; Rv2436) and *ask* (aspartokinase AsK; Rv3709c). The *aroK* gene (encoding Shikimate Kinase) was obtained from the laboratory of Chris Abell, University of Cambridge. The genes were amplified from H37Rv genomic DNA using the oligonucleotide primers shown in Table 3, and the PCR products subcloned into the selected pET vector (Novagen, Germany) using the applicable restriction enzymes.

**Table 3:** Forward and reverse primers used to amplify specific kinase genes. Note also preferred vector for each construct

Kinase	Gene and Rv identifier	Forward and Reverse primers (5′ → 3′)	Restri-ction Enzyme Recogni-tion site (underli-ned)	Selected pET vector
NDK	*ndkA* Rv2445c	*ndkA*-Fwd		pET-16b
		GGCATATGACCGAACGGACTCTGGTACTG	*Nde*I	
		*ndkA*-Rev		
		GTGGATCCTTAGGCGCCGGGAAACCAG	*Bam*HI	
HSK	*thrB* Rv1296	*thrB*-Fwd		pET-20a
		GGCATATGGTGACTCAAGCATTG	*Nde*I	
		*thrB*-Rev		
		GTCTCGAGACCGGGAACTCTTACTG	*Xho*I	
AK	*ackA* Rv0409	*ackA*-Fwd		pET-16b
		GGCATATGGAGTAGCACCGTGCTGGTGATCAA	*Nde*I	
		*ackA*-Rev		
		GTGGATCCTTACGCTCGGCGTCCGCCCAG	*Bam*HI	
GK	*glpK* Rv3696c	*glpK*-Fwd		pET-16b
		(GGCATATGTCCGACGCCATCCTAG	*Nde*I	
		*glpK*-Rev		
		CATGTCGACTTAGGACACGTCAACCCAATCC	*Sal*I	
ThiL	*thil* Rv2977c	*thil*-Fwd		pET-28a
		GGTACATATGACCACTAAAGATCACTC	*Nde*I	
		*thil*-Rev		
		GATCTCGAGTTACCCTAGCGAACCTTG	*Xho*I	
RBKS	*rbks* Rv2436	*rbks*-Fwd		pET-28a
		GTACATATGGCAAACGCCAGTGAG	*Nde*I	
		*rbks*-Rev		
		GATCTCGAGTTATGAACCGTTGTG	*Xho*I	
AsK^a^	*ask* Rv3709c	*ask* alpha-Fwd		pCDF-
		GATTACATATGGCGCTCGTCGTGCAG	*Nde*I	Duet-1
		*ask* alpha-Rev		(alpha)
		GATGTCGACTTACCGTCCCGTCCCCG-3’	*Sal*I	
		*ask* beta-Fwd		pET-26a
		GATCATATGGAAGACCCCATCCTGACCG	*Nde*I	(beta)
		*ask* beta-Rev		
		GCCCCTGCCCTGCCCAGCTGTATG	*Sal*I	
SK	*aroK* Rv2539c	Primers were not designed as the *aroK* plasmid was received from the laboratory of Chris Abell, University of Cambridge		pET-15b

^a^ AsK consists of 2 hetero-monomers (alpha and beta), to be co-expressed

### Expression of Mtb kinases

*E. coli* BL21(DE3) (Novagen, USA) was used as production host. For AsK production, two co-transformed plasmids were used for co-expression of the alpha and beta monomers. The recombinant strains were cultivated in 250 ml LB broth supplemented with the appropriate antibiotic(s) at 37 °C with shaking at 200 rpm and, at OD_600_ ~ 0.6, induced with 1 mM Isopropyl β-D-1-thiogalactopyranoside (IPTG) and incubated overnight at 28 °C. Production of SK was carried as described by Kenyon et al. [10]. The cells were harvested by centrifugation at 4080 *g* for 10 min at 4 °C.

### Purification of Mtb kinases

The biomass pellets were resuspended in 20 ml Binding Buffer (1 M NaCl, 20 mM Tris-HCl and 5 mM Imidazole: pH 7.9), lysed by sonication and re-centrifuged to separate the soluble and insoluble fractions.

The soluble kinases NDK, ThiL, RBKS, AsK and SK were purified using the Profinia™ Affinity Chromatography Protein Purification System (Bio-Rad, USA) with a 1 ml column containing nickel-iminodiacetic acid (Ni-IDA) resin. The Standard Native conditions and protocols were followed according to the manufacturer’s instructions. For AK and GK, MagReSyn™ NTA (ReSyn™ Biosciences. South Africa) was used for purification. The manufacturer’s scaled-up protocol was followed. The eluates were dialysed overnight in each selected dialysis buffer (Table 4).

**Table 4:** Dialysis buffers used for each Mtb kinase

Kinase	Dialysis buffer
NDK	50 mM Tris pH 8.0
	100 mM KCl
	1 mM DTT
	1 mM MgCl_2_
HSK	50 mM MOPS pH 8.0
	150 mM NaCl
	1 mM DTT
	10 mM MgCl_2_
AK and GK	50 mM Tris pH 7.5
	150 mM NaCl
	1 mM DTT
	5 mM MgCl_2_
ThiL and RBKS	50 mM MOPS pH 7.6
	150 mM NaCl
	1 mM DTT
	10 mM MgCl_2_
AsK	50 mM Tris pH 6.0
	200 mM NaCl
	1 mM DTT
	10 mM MgCl_2_
SK	50 mM Tris pH 7.5
	1 M NaCl

HSK was insoluble and was purified using an ÄKTA Avant (GE Healthcare, USA). The biomass pellet was resuspended in 40 ml Denaturation Solublisation Buffer (DSB; 50 mM NaH_2_PO_4_, 300 mM NaCl, 8 M urea; pH 7.9) and incubated for 2 h at 37 °C with shaking at 50 rpm. This was then lysed by sonication and centrifuged at 4080 g for 10 min at 4 °C. The supernatant was clarified through a 0.45 μm syringe filter and loaded onto a 25 ml bed volume Ni-NTA (nickel-nitrilotriacetic acid) column on the ÄKTA Avant, pre-equilibrated with DSB. After loading, the column was washed with DSB, and HSK was refolded on the column using a linear gradient from 100% DSB to 100% of the urea-free Lysis Equilibration Buffer (50 mM NaH_2_PO_4_, 300 mM NaCl; pH 7.9) before being eluted off the resin using Elution Buffer (50 mM NaH_2_PO_4_, 300 mM NaCl, and 250 mM Imidazole; pH 8.0). The eluate was dialysed overnight in HSK’s selected dialysis buffer (Table 4), and concentrated five-fold through a Vivaspin 10 kDa MWCO column (Sartorius).

The concentration of the proteins was determined using the Qubit® 2.0 Fluorometer (Life Technologies. USA) and Qubit Protein Assay Kit, as recommended by the manufacturer. A volume of 50 μl of all proteins except AK and GK, were snap-frozen in liquid nitrogen and stored at − 80 °C until assayed. For AK and GK, aliquots of 50 μl of the dialysed protein were mixed with 50% [v/v] glycerol before storage at − 80 °C.

### Determination of enzyme activity

The kinase samples were thawed on ice prior to setting up the enzyme assays. The HPLC assay reactions were carried out in 100 μl volumes and incubated at 37 °C. The assay was carried out as described by [10]. Briefly, the assay reactions consisted of 90 μl of the prepared reaction mixture (Table 5) with either 10 μl of enzyme, prepared in triplicate or 10 μl distilled water, prepared in duplicate, which served as a control blank. A range of ATP concentrations was assayed, with ATP and MgCl_2_ concentrations always kept at a 1:1 ratio [24]. After the pre-determined reaction time (Table 5), the reactions were stopped with 5% [v/v] 200 mM EDTA.2Na.2H_2_O and subsequently loaded onto an Agilent 1100 HPLC to measure the adenosine diphosphate (ADP) product formation and the reduction of the ATP substrate. The HPLC automatically injected 0.2 μl of each sample reaction mix onto a Phenomenex 5 μ LUNA C18 column with the mobile phase containing PIC A® (Waters Corporation. USA), 250 ml acetonitrile and 7 g KH_2_PO_4_ per litre of water. The flow rate of the mobile phase was 1 ml/min and the separated reactants were detected using a UV detector to measure absorbance at a wavelength of 259 nm. AMP, ADP and ATP standards were used to calibrate the HPLC and the levels of ADP in each sample were determined by using Agilent ChemStation (Revision B.02.01) software (Agilent Technologies. USA). Absorbance values obtained for the control containing distilled water were subtracted from the enzyme reactions. Favorable enzyme activity, in this study, was defined by achieving linearity to demonstrate a constant rate, as well as attaining percentage conversions (of ATP to ADP) within the range of 5–15%.

**Table 5:** Details of assay reactions for determination of enzyme activity and inhibition assays in the presence of various dilutions of P. sidoides extract

Enzyme	Enzyme Activity Assay reaction mixtures	*P. sidoides* inhibition Assay reaction mixtures	Incubation time
NDK	100 mM K-PO_4_ buffer (pH 6.8), 250 mM KCl 5 nM enzyme, 0.2 M Thymidine diphosphate	100 mM K-PO_4_ buffer (pH 6.8), 250 mM KCl 5 nM enzyme, 0.2 M Thymidine diphosphate	40 mins
HSK	50 mM HEPES buffer (pH 7.0), 450 mM KCl 704 nM enzyme, 10 mM Homoserine	50 mM HEPES buffer (pH 7.0), 450 mM KCl 704 nM enzyme, 10 mM Homoserine	4 h
AK	100 mM Tris buffer (pH 7.0), 250 mM KCl 223 nM enzyme, 10 mM Na-acetate	100 mM Tris buffer (pH 7.0), 250 mM KCl 223 nM enzyme, 10 mM Na-acetate	24 h
GK	100 mM Tris buffer (pH 7.0), 250 mM KCl 208.6 nM enzyme, 100 mM Glycerol	100 mM Tris buffer (pH 7.0), 250 mM KCl 208.6 nM enzyme, 100 mM Glycerol	24 h
ThiL	100 mM Tris buffer (pH 8.0), 250 mM KCl 2074 nM enzyme, 1 mM Thiamine monophosphate	100 mM Tris buffer (pH 8.0), 250 mM KCl 2074 nM enzyme, 1 mM Thiamine monophosphate	5 h
RBKS	100 mM Tris buffer (pH 7.2), 100 mM KCl 250 nM enzyme, 10 mM d-ribose	100 mM Tris buffer (pH 7.2), 100 mM KCl 250 nM enzyme, 10 mM d-ribose	4 h
AsK	100 mM Tris-HCl buffer (pH 7.5), 178.2 nM enzyme 10 mM l-Aspartic acid	100 mM Tris-HCl buffer (pH 7.5), 178.2 nM enzyme 10 mM l-Aspartic acid	6 h
SK	100 mM K-PO_4_ buffer (pH 6.8), 500 mM KCl 10 nM enzyme, 8 mM shikimic acid	100 mM K-PO_4_ buffer (pH 6.8), 500 mM KCl 10 nM enzyme, 8 mM shikimic acid	20 mins

### *Pelargonium sidoides* inhibitory activity

The Mtb kinases were thawed on ice. A 100 mg/ml stock solution of the *P. sidoides* crude extract was prepared in distilled water. A 10-fold serial dilution of the aqueous plant extracts, ranging from 1 × 10^1^ mg/ml to 1 × 10^− 5^ mg/ml, was then prepared before being stored at − 20 °C. The inhibitory activity determination was carried out using HPLC enzyme assays as described earlier, at a single ATP and MgCl_2_ concentration (1 mM each), and with the addition of varying concentrations of the *P. sidoides* plant extract. A water-only control was run in parallel, to serve as a negative control for activity comparison analysis. The reaction mixtures of each kinase (Table 5) were incubated at 37 °C for a specific time and thereafter stopped with 5% [v/v] 200 mM EDTA.2Na.2H_2_O. The reactions were subsequently analysed as above. All assays were carried out in triplicate and the standard deviation determined and plotted as part of the data. The IC_50_ values were calculated, with the aid of GraphPad Prism 5 (GraphPad Software Inc. USA) as specified by the software when plotting log [Inhibitor] concentration versus the enzyme activity.

Enzyme acticvity assays contained 0.25–1.5 mM ATP and 2 mM MgCl_2_. Dose response assays contained 0–1 mg/ml *P. sidoides* plant extract, 1 mM ATP and 1 mM MgCl_2_.

### Protein expression validation

Each purified protein was then proteolytically fragmented using the Thermo Scientific™ SMART Digest™ kit as per the manufacturer’s instructions. The peptide fragments were lyophilized and made up in 20 μl 2% v/v acetonitrile containing 0.1% v/v formic acid for mass spectroscopy analysis.

#### Liquid chromatography (Dionex nano-RSLC)

Liquid chromatography was performed on a Thermo Scientific Ultimate 3000 RSLC equipped with a 5 mm × 300 mm C_18_ trap column (Thermo Scientific) and a CSH 25 cm × 75 μm 1.7 μm particle size C_18_ column (Waters) analytical column. The solvent system employed was loading: 2% acetonitrile:water; 0.1% formic acid; Solvent A: 2% acetonitrile:water; 0.1% formic acid and Solvent B: 100% acetonitrile:water, 0.1% formic acid. The samples were loaded onto the trap column using loading solvent at a flow rate of 10 μL/min from a temperature controlled autosampler set at 7 °C. Loading was performed for 5 min before the sample was eluted onto the analytical column. Flow rate was set to 325 nL/minute and the gradient generated as follows: 2.0 -10% B for 4 min; followed by 10–35% B from 4 to 60 min and finally 35–50% B from 60 to 70 min. Chromatography was performed at 40 °C and the outflow delivered to the mass spectrometer through a stainless steel nano-bore emitter.

#### Mass spectrometry

Mass spectrometry was performed using a Thermo Scientific Fusion mass spectrometer equipped with a Nanospray Flex ionization source. The sample was introduced through a stainless steel emitter. Data was collected in positive mode with spray voltage set to 1.8 kV and ion transfer capillary set to 280 °C. Spectra were internally calibrated using polysiloxane ions at m/z = 445.12003 and 371.10024. MS1 scans were performed using the orbitrap detector set at 120000 resolution over the scan range *m/z* = 350–1650 with Adaptive Gain Control (AGC) target at 5 × 10^4^ and maximum injection time of 40 ms. Data was acquired in profile mode.

MS2 acquisitions were performed using monoisotopic precursor selection for ion with charges + 2 − + 7 with error tolerance set to +/− 10 ppm. Precursor ions were excluded from fragmentation once for a period of 60 s. Precursor ions were selected for fragmentation in High Energy Dissociation (HCD) mode using the quadrupole mass analyser with HCD energy set to 30%. Fragment ions were detected in the orbitrap mass analyzer set to 15,000 resolution. The AGC target was set to 5 × 10^4^ and the maximum injection time to 30 ms. These data was acquired in centroid mode.

The raw files generated by the mass spectrometer were imported into Proteome Discoverer v1.4 (Thermo Scientific) and processed using the Sequest algorithm. Database interrogation was performed against a concatenated database created using the Uniprot *M. tuberculosis* database with the cRAP contaminant database. Semi-tryptic cleavage with 2 missed cleavages was allowed for. Precursor mass tolerance was set to 10 ppm and fragment mass tolerance set to 0.05 Da. Demamidation (arginine and glutamine), oxidation (methionine) and acetylation of protein N-terminal was allowed as dynamic modifications and thiomethyl of cysteine as static modification. Peptide validation was performed using the Target-Decoy PSM validator node. The output files from Proteome Discoverer were imported in to Scaffold Q+ and the assignments validated using X1Tandem and the PeptideProphet and ProteinProphet algorithms.

## Supplementary information

**Additional file 1 **SDS-PAGE gels of the Mtb his-tagged kinases purified from *E. coli* BL21 (DE3). A) Nucleotide diphosphate kinase. B) Histidine kinase. C) Acetate kinase and Glycerol kinase. D) Thiamine monophosphate kianse (T) and Ribokinase (R). E) Aspartokinase. F) Skikimate kinase. Protein concentrations of purified enzymes.

## Data Availability

The datasets used and/or analysed during the current study are available from the corresponding author on reasonable request.

## Abbreviations

Mtb: *Mycobacterium tuberculosis*
NDK: Nucleoside diphosphokinase
HSK: Homoserine kinase
AK: Acetate kinase
GK: Glycerol kinase
ThiL: Thiamine monophosphate kinase
RBKS: Ribokinase
AsK: Aspartokinase
SK: Shikimate kinase
IPTG: mM Isopropyl β-D-1-thiogalactopyranoside
