# Supplementary material for: Novel kinase platform for the validation of the anti-tubercular activities of Pelargonium sidoides (Geraniaceae)
Source: BMC Biotechnol. 2020 Sep 29;20:50. doi: 10.1186/s12896-020-00643-w (PMC7523293; doi:10.1186/s12896-020-00643-w)

**Supplementary Information**

Protein fractions using the Ni-IDA resin on the Profinia™ purification system. Also included are typical enzyme protein concentration.

**M**

**Total protein**

**Insoluble**

**Loaded**

**Flowthrough**

**Wash1**

**Wash 2**

**Eluate**

| 1. Nucleotide diphosphate kinase   **250**  **130**  **100**  **\\\\**  **70**  **55**  **35**  **25**  **15**  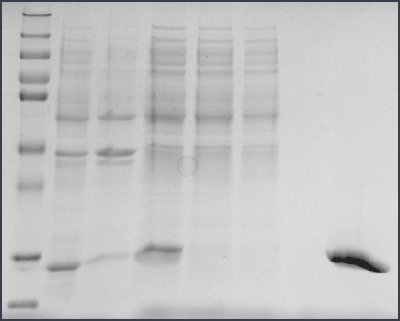  M  Loaded  Flowthrough  Wash 1  Wash 2  Eluate 1  Eluate 2  Eluate 3  14.4 |
| --- |
| 1. Histidine Kinase   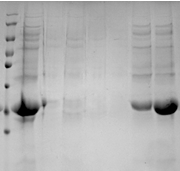  **250**  **130**  **100**  **\\\\**  **70**  **55**  **35**  **25**  **15**  M  AK Loaded  AK Flowthrough  AK Wash 1  AK Wash 2  AK Wash 3  AK Eluate  GK Loaded  GK Flowthrough  GK Wash 1  GK Wash 2  GK Wash 3  GK Eluate  33.4 |
| 1. Acetate and glycerol kinase   **250**  **130**  **100**  **\\\\**  **70**  **55**  **35**  **25**  **15**  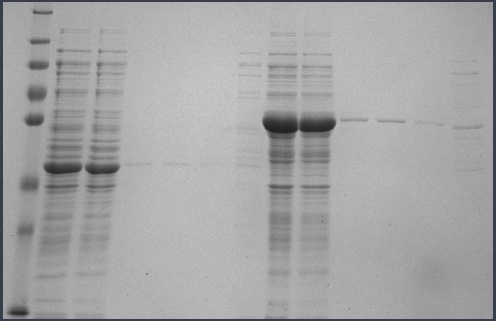  43.7 AK  58.2 GK |

SDS-PAGE gels of the Mtb his-tagged kinases purified from *E. coli* BL21 (DE3).. A) Nucleotide diphosphate kinase. B) Histidine kinase. C) Acetate kinase and Glycerol kinase. M = (PageRuler™ Plus Pre-stained Protein Ladder, Fermentas) and the sizes of this marker are indicated to the left of the gels. The blue arrows actual protein masses in kDA.

| D) Thiamine monophosphate kinase  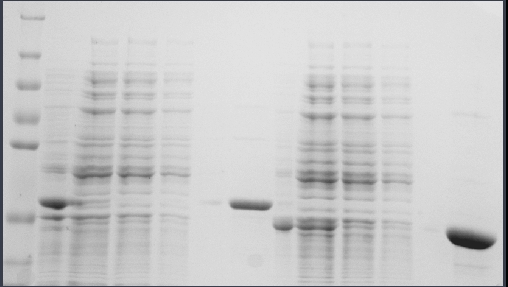  M  Insoluble  Loaded  Flowthrough  Wash 1  Wash 2  Eluate  **250**    **130**  **100**  **\\\\**  **70**  **55**  **35**  **25**  32.3 RBKS  36.4 ThiL |
| --- |
| 1. Aspartokinase   **250**  **130**  **100**  **\\\\**  **70**  **55**  **35**  **25**  **15**  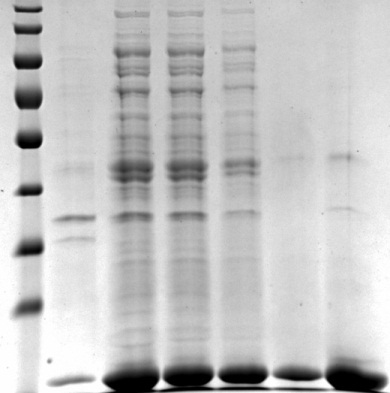  M  Loaded  Flowthrough  Wash 1  Wash 2  Eluate  18 (beta)  44.6 (alpha) |
| 1. Shikimate kinase   **250**  **130**  **100**  **\\\\**  **70**  **55**  **35**  **25**  **15**  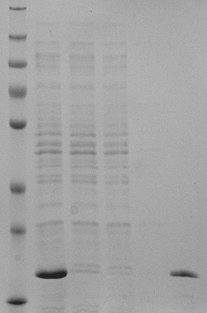  20.7 |

SDS-PAGE gels of the Mtb his-tagged kinases purified from *E. coli* BL21 (DE3). D) Thiamine monophosphate kianse (T) and Ribokinase (R). E) Aspartokinase. F) Skikimate kinase. M = represents the molecular mass marker (PageRuler™ Plus Pre-stained Protein Ladder, Fermentas. USA) The blue arrows actual protein masses in kDA.

M

Insoluble

T Loaded

T Flowthrough

T Wash 1

T Wash

2 Eluate

R Insoluble

R Loaded

R Flowthrough

R Wash 1

R Wash 2

R Eluate

###

**Determination of Protein Quantitation**

The Qubit® 2.0 Fluorometer quantitated the purified proteins swiftly and accurately. This molecular technique provided a simple way to determine the concentration of the available protein for further analysis. The results are presented in Table below.

Concentration of purified kinase proteins (presented in µg/ml)

| Kinase | Enzyme Concentration (µg/ml) |
| --- | --- |
| NDK | 1480 |
| HSK | 935 |
| AK | 390 |
| GK | 348 |
| ThiL | 755 |
| RBKS | 800 |
| AsK | 1128 |
| SK | 2030 |

The use of sequential purification techniques were intended to generate smaller amounts of purer active protein. The concentration levels of protein expression obtained through these sequential protein purification techniques and systems were suitable for further functional analysis and screening protocols.

**ACETATE KINASE**

**SEQUENCE** (K)MLAEDGIDLQTcGLVAVGHR **Peptide Identification Probability 99.7%**

**SEQUEST XCorr** 4.273295 **Seq Delta Cn** 0.6725566 **X! Tandem** 9.130768

**Modification.** Carbamidomethyl **Unique peptides** 34 **Unique spectra** 47
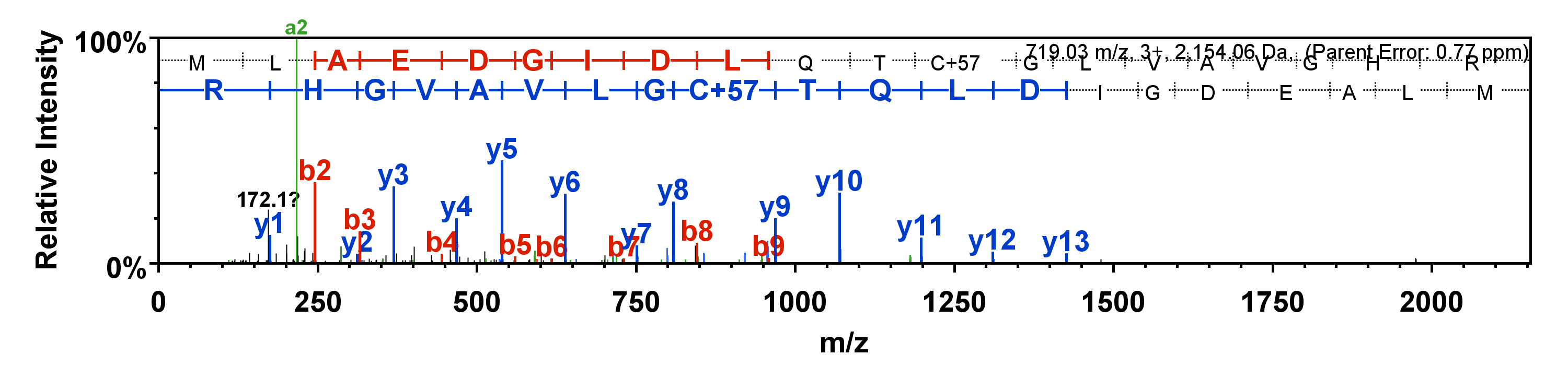


**GLYCEROL KINASE**

**SEQUENCE** (R)DQLGIISGAAQSEALAR(Q) **Peptide Identification Probability** 99.7%

**SEQUEST XCorr** 4.122187**Seq Delta Cn** 0.5792115**X! Tandem** 10.721247

**Modification.** None **Unique peptides** 55 **Unique spectra** 68


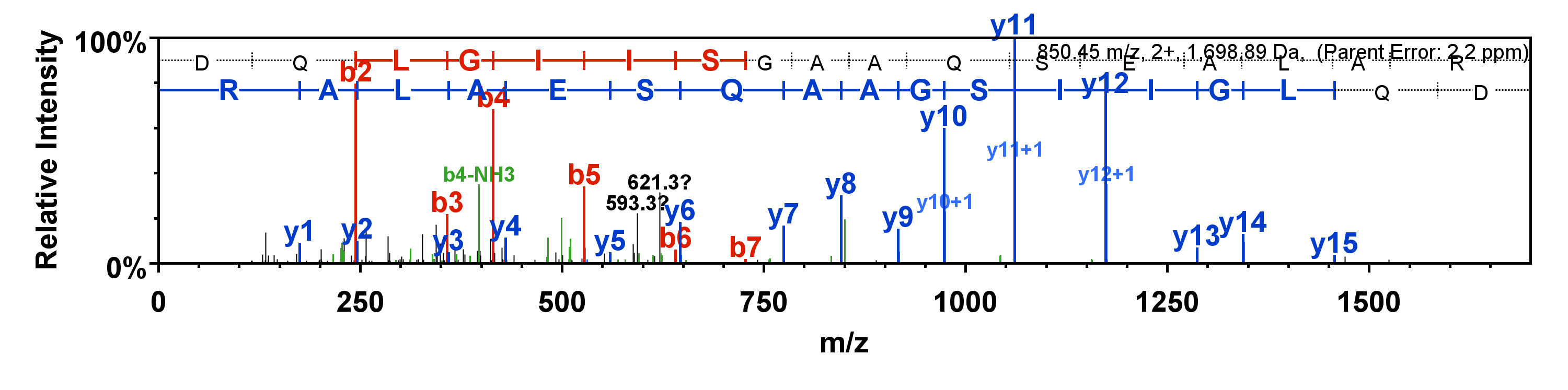


**HOMOSERINE KINASE**

**SEQUENCE** (K)GFAVTELTVGEAVR(W) **Peptide Identification Probability** 100%

**SEQUEST XCorr** 3.887441 **Seq Delta Cn** 0.6896717 **X! Tandem** 8.721247

**Modification.** None **Unique peptides** 65 **Unique spectra** 100


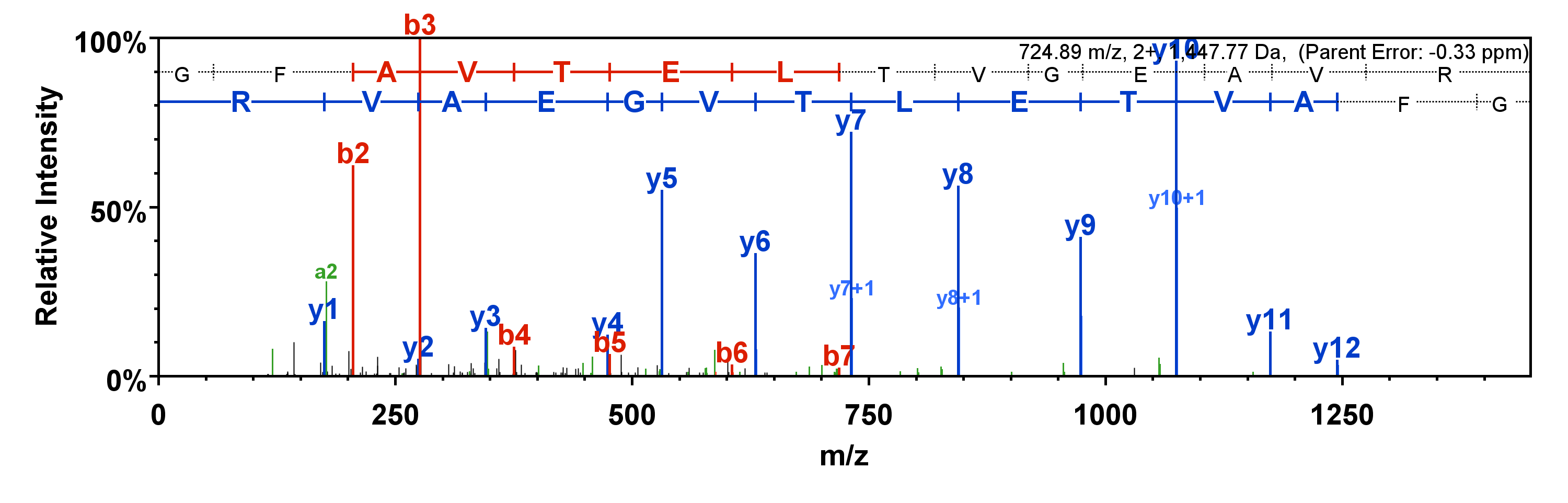


**NUCLEOSIDE diPHOSPHATE KINASE**

**SEQUENCE** (R)KGLTIAALQLR(T) **Peptide Identification Probability** 100%

**SEQUEST XCorr** 3.86 **Seq Delta Cn** 0.69 **X! Tandem** 3.11

**Modification.** None **Unique peptides** 11 **Unique spectra** 18


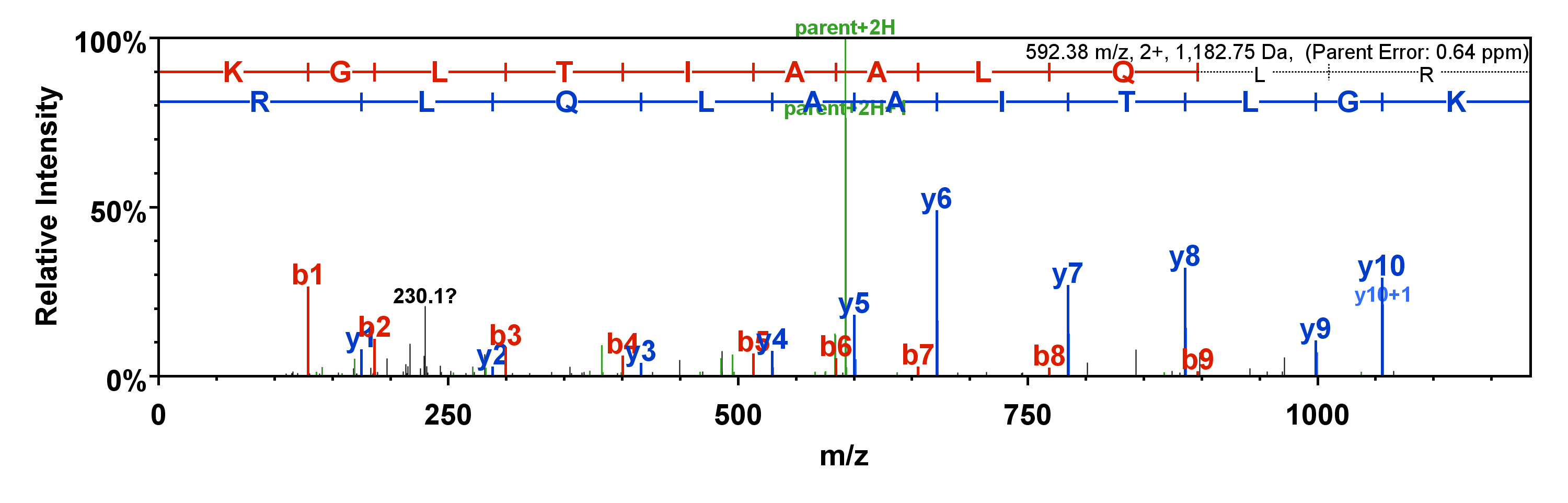


**THIANINE MONOPHOSPHATE KINASE**
**SEQUENCE** (R)TVVSTDMLVQDSHFR(L) **Peptide Identification Probability** 100%

**SEQUEST XCorr** 1.8 **Seq Delta Cn** 0.487 **X! Tandem** 2.89

**Modification.** None **Unique peptides** 3 **Unique spectra** 3


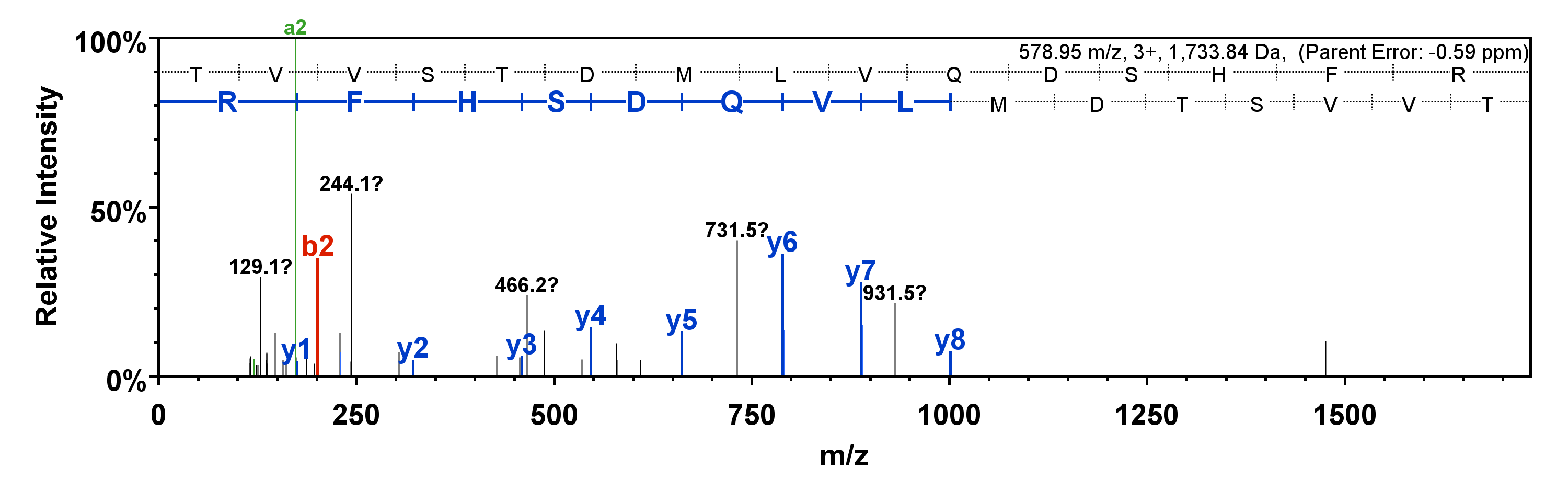


**ASPARTOKINASE**

**SEQUENCE** (R)cVEYARRHnIP(V) **Peptide Identification Probability** 99.7%

**SEQUEST XCorr** 0.43 **Seq Delta Cn** -0.1 **X! Tandem** 0

**Modification.** None **Unique peptides** 3 **Unique spectra** 3


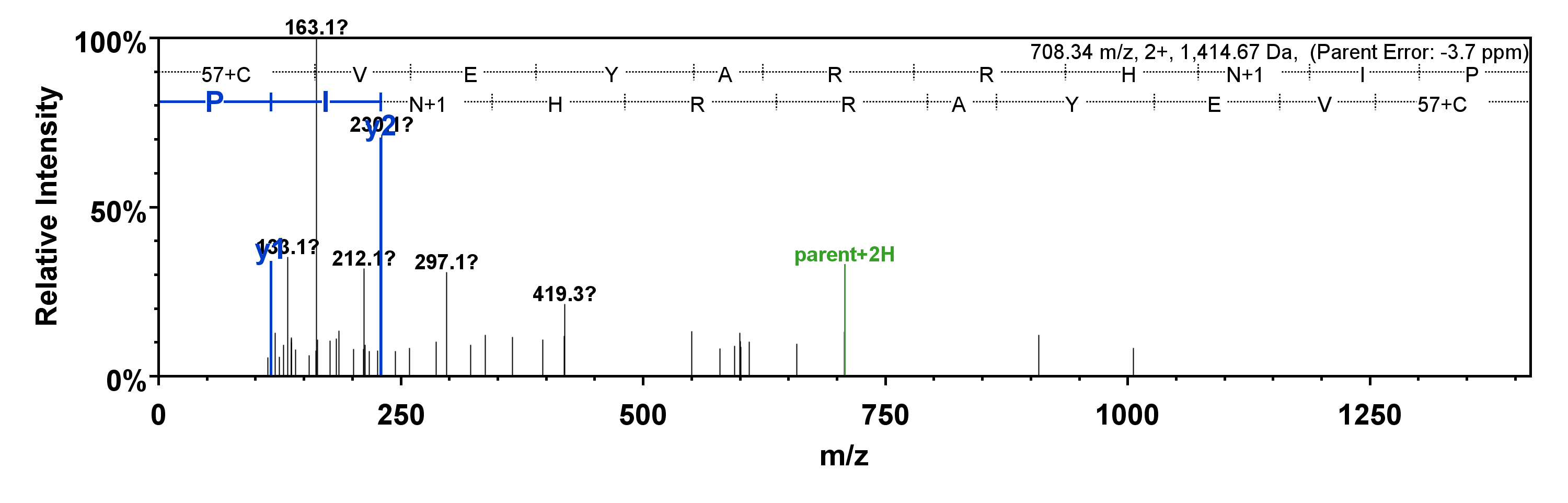

Supplement: Supplementary file 1 — Additional file 1 SDS-PAGE gels of the Mtb his-tagged kinases purified from E. coli BL21 (DE3). A) Nucleotide diphosphate kinase. B) Histidine kinase. C) Acetate kinase and Glycerol kinase. D) Thiamine monophosphate kianse (T) and Ribokinase (R). E) Aspartokinase. F) Skikimate kinase. Protein concentrations of purified enzymes. [file 12896_2020_643_MOESM1_ESM.docx]
